# Predicting groundwater storage from seasonal managed aquifer recharge: insights from machine learning and explainable AI techniques

**DOI:** 10.1007/s12665-026-12825-4

**Published:** 2026-02-18

**Authors:** Valdrich J. Fernandes, Perry G. B. de Louw, Coen J. Ritsema, Ruud P. Bartholomeus

**Affiliations:** 1 Soil Physics and Land Management Group, Wageningen University & Research, Wageningen, The Netherlands; 2 Department of Groundwater and Water Security, Deltares, Utrecht, The Netherlands; 3 KWR Water Research Institute, Nieuwegein, The Netherlands

**Keywords:** Machine learning, Managed Aquifer recharge, Interpretable AI, Scenario optimisation, Drought mitigation

## Abstract

Managed Aquifer Recharge (MAR) is widely used to enhance groundwater storage and support sustainable water use. To support site selection and planning, Machine Learning (ML) models are increasingly used as computationally efficient surrogates for traditional numerical models. While ML has shown promise for steady-state simulations, capturing transient responses remains a challenge, yet these are essential for understanding how recharged water is retained through dry periods. In this study, we use ML to model transient MAR effects by decomposing the groundwater storage time series after recharge ceases into two components: the MAR-response and a decay coefficient, assuming exponential storage decline. This simplified representation captures long-term storage dynamics, with U-Net and XGBoost accurately predict these components (R^2^ > 0.82) for the Baakse Beek catchment in the sandy, drought-sensitive soils of the Netherlands. The trained models are computationally efficient, making large-scale scenario testing and optimization feasible. Explainable AI techniques, specifically SHAP values, were used to identify key site management decisions and surface water properties that control MAR effectiveness. These findings illustrate the potential of explainable AI and ML surrogate models to enhance the planning and optimization of MAR.

## Introduction

Seasonal water availability has increasingly become a concern worldwide. Even countries with typically robust water systems have recently faced periods of water stress during extended periods of droughts. The 2018-2020 drought, for instance, affected most of continental Europe, limiting surface water availability in major international rivers (Rakovec et al. 2022). Similar extended periods of droughts are expected to become more frequent in future climatic conditions (Gu et al. 2023; Aalbers et al. 2023; Hari et al. 2020; van der Wiel et al. 2021). The 2018 drought saw a general deterioration of surface water quality, further exacerbating the stress on water users dependent on these sources (Wolff and van Vliet 2021). This combination of poor water quality and scarcity is expected to worsen in Western Europe, particularly between June and October (Jones et al. 2024). Capturing excess water during the seasonal surplus could help mitigate some of the demand during the dry season.

Addressing this vulnerability requires prioritizing water management solutions that increase summer water availability (Bartholomeus et al. 2023), while still accounting for risks of floods and waterlogging. Efficient groundwater management could address some of the vulnerability by serving as a buffer, capturing water during surplus that supports demands during dryer periods. While much of this recharge occurs naturally, it can be further enhanced by recharging surplus water into the groundwater aquifers using Managed Aquifer Recharge (MAR) techniques (Dillon et al. 2020, 2019; Hartog and Stuyfzand 2017). The effectiveness of MAR is dependent on many inter-related factors such as slope, soil type, aquifer properties, depth to groundwater, and drainage intensity. Studies often rely on techniques such as Multi-Criteria Decision Analysis (MCDA) to help identify potential MAR locations (Javadi et al. 2021; Malekmohammadi et al. 2012; Sallwey et al. 2019; Sandoval and Tiburan 2019). MCDA is a family of methods that structure decisions with multiple criteria and combine evidence with stakeholder preferences to rank or select among alternatives (Belton and Stewart 2002; Huang et al. 2011). However, Sallwey et al. (2019) showed that there is a large variation in the criteria used to identify optimal MAR sites. A more thorough analysis would require comparing multiple potential locations for MAR for various recharge rates using a detailed numerical groundwater model.

Maples et al. (2020) attempted to identify the key criteria affecting MAR effectiveness through local and global sensitivity analysis. While the local sensitivity was performed on 30 days of recharge and 90 days without, the global sensitivity was performed on only 10 days of recharge to manage computational demands. They also focused on the hydraulic conductivity and a geologic proxy parameter. This approach effectively identified the key criteria affecting MAR effectiveness locally within the parameter range, near the calibrated settings, but the short window and two-parameter scope limit insight into the globally relevant key critera. Furthermore, their technique excludes interaction between the parameters. Machine Learning (ML) based surrogate modelling could be applied here to address the computational demand.

Surrogate modelling involved training a computationally efficient ML model on data from a computationally expensive model. A previous study demonstrated the use of U-Net as a surrogate model for estimating the steady-state groundwater response to managed aquifer recharge (Fernandes et al. 2024). The resulting surrogate reproduced the steady-state response in under 0.2 seconds, over 3,000 times faster than the original simulations, while maintaining high predictive accuracy. However, optimizing the steady-state MAR response does not account for the transient behavior of MAR, as water is recharged during periods with a water surplus, to buffer the water availability using the subsurface. The first goal of this paper therefore is to investigate a potential technique for ML to capture the transient dynamics of groundwater response to MAR, capturing both immediate effects and longer-term dynamics during periods without recharge.

In addition to capturing the transient behavior of groundwater storage due to MAR in ML, this study focuses on model explainability. Surrogate modelling technique has recently been applied for transient MAR-scenarios, where recharge was applied during high precipitation events (Dai et al. 2025) using seven ML models including CNN3d (a convolution neural network with three hidden 3D convolution layers) and ViT3d (Dosovitskiy et al. 2021) that were identified to be more interpretable. In terms of computational efficiency, the trained ML models were six orders of magnitude faster than the numerical groundwater model. Even when including the training time, the ML model was two orders of magnitude faster at generating 500 simulations. However, they identified interpreting their ML models to be complex and cumbersome.

This challenge can be addressed by applying explainable AI techniques such as SHapley Aditive exPlanations (SHAP) on a simpler and more explainable ML model (Lundberg and Lee 2017). SHAP quantifies the contribution and direction of influence of each input feature on model predictions, offering an interpretable view of the relations identified by the model. By aggregating these feature contributions across the entire dataset, SHAP reveals generalized input–output relationships that extend beyond the local scope of local sensitivity analyses and can form a foundation for MCDA.

This study presents ML surrogate models to efficiently and accurately predict the transient dynamics of groundwater response to MAR, capturing both the recharging process of MAR during periods of water surplus and the decay or discharge of the stored water volume during periods without recharge. Further, SHAP values are used to identify and quantify the influence of each geo-hydrological input on the groundwater response, providing explainable insights into the model’s prediction. These insights not only enhance our understanding of the system but also lay groundwork for integrating MCDA to identify potential locations for MAR. Through this approach, this study aims to develop a comprehensive understanding of the key criteria influencing water storage due to MAR in the short to medium-term period.

## Methodology

### General overview

**Fig. 1:**
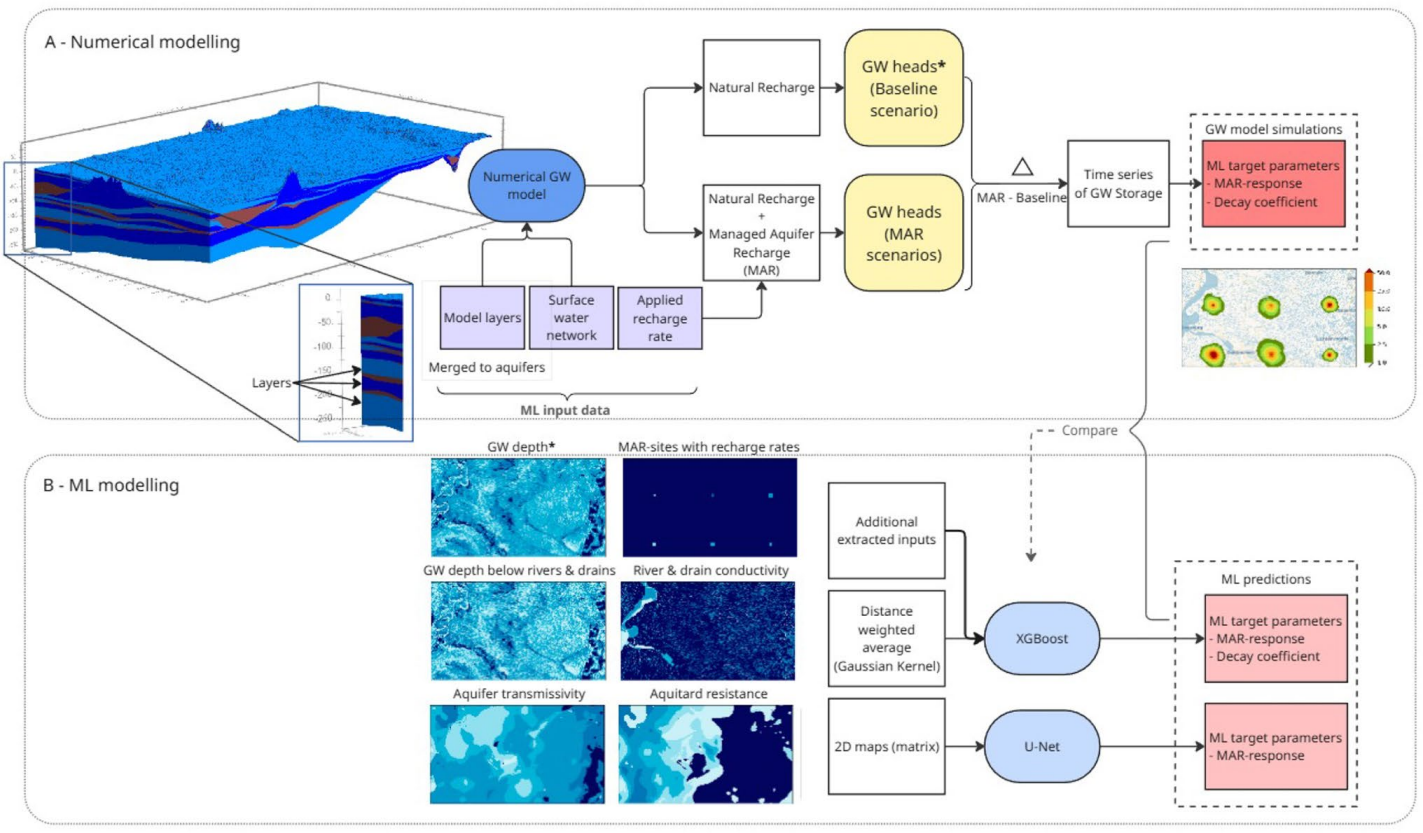
Schematic overview of the methodology. (**A**) Numerical groundwater (GW) model used to simulate MAR sites and compute the resulting increase in groundwater storage. (**B**) Machine-learning framework, showing the two ML models trained to reproduce the outputs of A using inputs derived from the numerical GW model. The input maps used for the U-Net model are also shown in panel B. Groundwater depth (marked with an asterisk, *) and specific yield (not shown, as it is not an input to U-Net) are obtained from the baseline (natural recharge) scenario

The methodology employed in this study follows a typical workflow for surrogate modelling, where the groundwater response to hypothetical MAR sites are simulated with a numerical groundwater model, which makes up the training and testing data for the ML surrogate models (Fig. 1B) . The groundwater response is calculated as the difference in the groundwater heads between scenarios with MAR and a scenario with only natural recharge (Fig. 1A). The location, size, and recharge rate are randomly selected to represent the entire range of possibilities within the demonstration region. The surrogate modelling technique is applied to the Baakse Beek catchment, situated in the eastern sandy soils of the Netherlands. This region is characterized by highly transmissive and freely draining soils with a dense surface water network of ditches and streams that drain the groundwater reservoir. This network has been significantly intensified over time, increasing the region’s vulnerability to water shortages during extended dry periods. The study examines how the volume of water stored by MAR decays toward natural groundwater levels during dry periods without recharge.

### Numerical groundwater model

The artificial recharge is simulated using a numerical groundwater model, AMIGO (Vreugdenhil 2021), which represents the subsurface in the part of Gelderland province that is under the jurisdiction of the water board Rijn en IJssel in the Netherlands. AMIGO is a coupled unsaturated-saturated zone model similar to those used in Querner et al. (2016), and van Walsum and Veldhuizen (2011) where the saturated zone is represented using MODFLOW-2005 (Harbaugh 2005) while the unsaturated zone is represented using MetaSWAP. Gridded meteorological data from the AMIGO dataset, including precipitation and Makkink reference crop evaporation, is applied with the MetaSWAP that calculates the effective groundwater recharge, which is then applied to the saturated zone model. The saturated zone represents a 200 m thick sequence of Pleistocene sands, which is underlain by the clayey Breda formation. This formation represents the model basement, represented in the model by a no-flow boundary condition.

The groundwater model was cropped to a 765 km^2^ rectangular extent that contains the Baakse Beek catchment. The model edges are represented by time-varying specified heads that were derived from a long-term simulation from April 2004. A buffer of 2.25 km was maintained between the model edges and the catchment boundary, ensuring that the hypothetical MAR sites were also at least 2.25 km from the model boundary. This minimizes the influence of the boundary conditions on the groundwater heads near the MAR sites. Stream and tile drainage were represented using the river (RIV) and drain (DRN) packages in MODFLOW-2005, respectively. A fixed river stage was used for all streams except for the river IJssel, where monthly average stage data were available. Furthermore, the infiltration from ephemeral streams in the region was reduced using an infiltration factor implemented in iMODFLOW (Vermeulen et al. 2021).

In the simulated scenarios, the artificial recharge is simulated using the recharge (RCH) package in MODFLOW (‘MAR sites with recharge rates’ in Fig. 1B). This recharge is applied directly to the saturated zone, bypassing the unsaturated zone. It represents the effective recharge rate that reaches the saturated zone and is analogous to aquifer recharge applied below the surface, such as through sub-surface irrigation. As a result, the recharged water does not influence the evapotranspiration, except due to the increase in the groundwater level. While this is a simplification of reality, this assumption has minimum impact on the system, especially away from the MAR site. At the recharge location itself, evaporation is likely underestimated, leading to an overestimation of the effective recharge volume.

### MAR scenarios

**Fig. 2:**
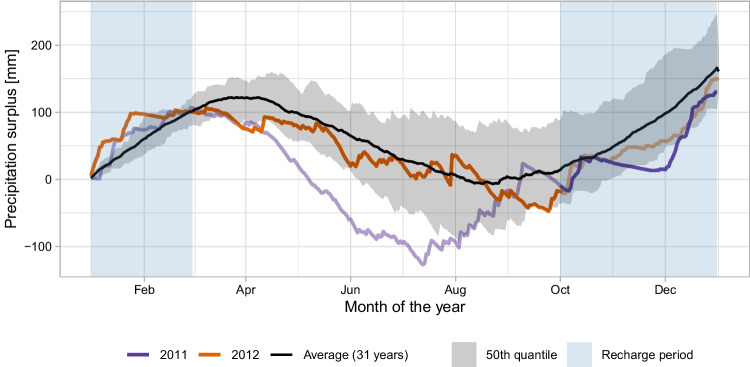
Cumulative potential precipitation surplus (i.e. precipitation minus reference evaporation according to Makkink 1957) in the simulated years, 2011 and 2012 as compared to the average surplus between 1993 and 2024. Artificial recharge is applied between 1^st^ October 2011 till 1^st^ March 2012, when precipitation exceeds the reference evaporation

In the scenarios considered, MAR is only applied in months when precipitation exceeds the evaporation demand for a year with average meteorological conditions. For this analysis, the meteorological data from the Hupsel station, data made available by the Royal Netherlands Meteorological Institute (KNMI), is analyzed as it is located within the study area (KNMI Data Platform 2014a, b). Complete data is available from October 1993 onwards based on which the annual cumulative evaporation excess precipitation is calculated. Based on the 31 years of available data, 2012 was selected as a representative year for average meteorological conditions, where the precipitation excess fell within the 25^th^ to 75^th^ percentile for most of the year (see Fig. 2). The cumulative precipitation surplus at Hupsel increased between October 1, 2011, and March 1, 2012 (blue shaded region in Fig. 2), indicating higher water availability for artificial recharge. In the scenarios considered, artificial recharge was simulated during this period of water surplus and the progression of the groundwater response during the period without artificial recharge was analyzed until October 1, 2012. In addition, a baseline scenario representing natural conditions was simulated, and the difference between the two scenarios was used to quantify groundwater storage due to MAR.

To ensure representative antecedent conditions for all scenarios, a two-year start-up run was simulated from 1^st^ October 2009 to 30^th^ September 2011. The initial groundwater levels for this start-up run were derived from a simulation starting in April 2004, which was conducted outside the scope of this study. Since initial soil moisture conditions for MetaSWAP were unavailable for this earlier run, conditions that ensure an average recharge flux were used as the starting conditions for the start-up run.

We simulated a total of 720 hypothetical artificial MAR sites, evenly spaced across the model domain, with 500 sites are used to train the surrogate models and 220 for evaluating its performance. The use of 720 sites was based on Fernandes et al. (2024), who demonstrated that this sampling density provides sufficient spatial coverage to train and evaluate the surrogate model effectively. The recharge rate at these sites ranged from 5 to 25 mm/day, and the site sizes varied between square sites of 0.1 and 1 km^2^. The recharge rate and the size of each site were selected randomly using Latin hypercube sampling technique. Recharge-site locations were selected using an orthogonal array design to achieve balanced coverage across the model domain. Six MAR sites were simulated simultaneously in each scenario to reduce the number of numerical groundwater model simulations needed (‘MAR sites with recharge rates’ in Fig. 1B). The interaction between the MAR sites was minimized by maintaining a separation distance of six times the leakage factor between the sites. The sites were split into a training and a testing dataset by random sampling, and these datasets were kept consistent across all ML model training process.

### Target parameter estimation

This study focuses on predicting the progression of groundwater storage during periods without MAR. Groundwater storage is defined as the total increase in groundwater heads, analogous to the volume of the aquifer saturated due to MAR. Rather than predicting the complete time series of the groundwater storage during periods without MAR, the response is decomposed into two components of the exponential decay function. The response peaks during the recharge period and then gradually returns to natural groundwater levels once recharge stops. Although groundwater systems involve non-linear interactions, simulation results (see Fig. 4) suggest that a simple exponential decay provides a parsimonious yet sufficiently accurate representation of storage decline. This behavior likely reflects the first-order, head-dependent outflow formulation in MODFLOW, in which discharge scales with increases in groundwater head, corresponding to changes in groundwater storage. Such behavior is commonly observed in systems where the rate of change is proportional to the current quantity. In hydrology, the discharge from a linear reservoir follows this behavior, which decreases exponentially after precipitation ends. In such reservoirs, the discharge $Q_t$ at time $t$ is directly proportional to the water storage $S_t$ within the catchment, governed by the reservoir constant $k$ (Wittenberg 1994; Pedersen et al. 1980; Dingman 2015). This relationship is captured in Eq. 1, which can be expressed in terms of the initial discharge in Eq. 2. Assuming no external fluxes like recharge or extraction, the storage dynamics can be derived as shown in Eq. 3.

In this analogy, the groundwater response to artificial recharge can be compared to the storage $S$ in a linear reservoir. The initial storage $S_0$ represents the MAR-response, or the groundwater response at the end of the recharge period d, while the reservoir constant $k$ serves as the decay coefficient, which defines the rate at which the groundwater storage is drained to the surface water network. Together they describe both the magnitude and the persistence of storage due to MAR, providing a clear understanding of how the system returns to equilibrium. These two parameters, the MAR-response and the decay coefficient, are the target parameters for the ML models. These terms are used throughout the paper to identify the two separate phases of MAR.

1 $$\begin{aligned} Q_t= & k \cdot S_t \end{aligned}$$

2 $$\begin{aligned} Q_t= & Q_0 \cdot e^{-k t}\end{aligned}$$

3 $$\begin{aligned} S_t= & S_0 \cdot e^{-k t} \end{aligned}$$

In this study, MAR is applied from 1^st^ September 2011 till 1^st^ March 2012 and simulations continue to 1^st^ October 2012 (219 days). The MAR-response is the groundwater storage on 1^st^ March 2012, while the decay coefficient is calculated from the remaining 219 days without MAR. For context, a decay coefficient, $k = - \frac{\log 0.2}{219} \approx 0.0073$ day^-1^ implies that the stored volume decays to 20 % of its MAR-response by 1^st^ October 2012 (see Eq. 3). Note the inverse relation: larger k corresponds to less long-term storage remaining at the end of the period. A high MAR-response and a low decay coefficient are desirable.

Both target parameters were derived from the numerical model simulation results, which provide spatially distributed groundwater heads, and thus storage, for each grid cell surrounding a MAR site. The MAR-response can be determined at the grid-cell scale, reflecting the spatial distribution of increases in groundwater storage at the end of recharge. In contrast, the decay coefficient represents the overall drainage behavior of the entire MAR site, summarizing the rate of storage loss after recharge ends. Accordingly, groundwater storage was aggregated across all grid cells surrounding a MAR site, and both parameters were calculated from the resulting time series (as seen on the right-hand side of Fig. 1A). While the MAR-response can also be derived from steady-state simulations, the decay coefficient is defined exclusively under transient conditions, as it captures the temporal dynamics of post-recharge storage and groundwater head.

**Fig. 3:**
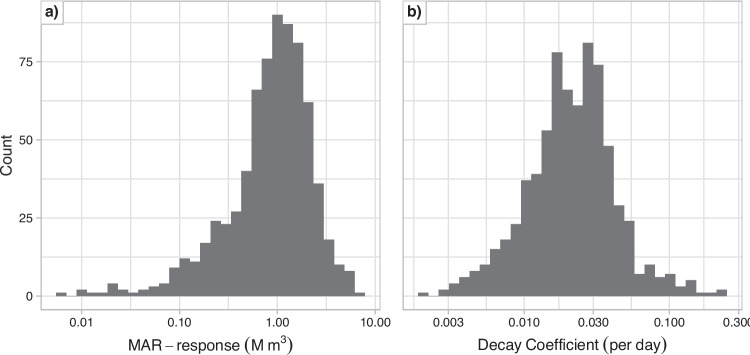
Distribution of the target variables, the MAR-response, Panel a, and the decay coefficient, Panel b, based on 720 hypothetical MAR sites simulated by the numerical groundwater model

The MAR-response is the increase in groundwater storage at the end of the recharge period and can be taken directly from the time series. In contrast, the decay coefficient is obtained by fitting a robust linear regression (rlm, *MASS* package in R) to the log-transformed storage time series after recharge ceases. Both target variables display right-skewed distributions. This skew would make the trained ML model biased to predict low values as these values are more common. To minimize the bias and make the target variables more normally distributed, the MAR-response and decay coefficient, are log-transformed, see Fig. 3. However, the target variables described in the figures and results of this study are back-transformed to the original scale.

**Table 1:** Models used in this study with their respective targets, simulation type, and indication of whether SHAP-based explanations were analyzed. The complete list of input features and their derivation is provided in “ML data preparation” section for both ML models

ML Target Parameter	Model	Scenario type^*1*^	SHAP implemented
MAR-response	Num. groundwater model^*2*^	T	−
	Num. groundwater model	S	No
	U-Net	T	No
	XGBoost	T	Yes
Decay coefficient	Num. groundwater model^*2*^	T	−
	XGBoost	T	Yes

^*1*^T – Transient scenarios; S – Steady-state scenarios

^*2*^Model used to generate training data

### ML procedure

#### ML models used

Two ML models were used to analyze the response to artificial recharge, U-Net and XGBoost. The models differ in their use of convolutional layers that allow U-Net to automatically learn and extract relevant features from the input data, reducing the need for manual feature engineering and improving predictive accuracy. Compared to tree-based models like XGBoost, these models are less interpretable, making it more challenging to verify the relations underlying their predictions. Comparison of the two models enables assessment of the trade-off between interpretability and predictive accuracy.

U-Net has previously been shown to accurately reproduce the steady-state response to artificial recharge (Fernandes et al. 2024). In this study, it is trained to estimate the transient MAR-response at the grid scale, capturing the spatial distribution of groundwater storage increases around each MAR site. In contrast, XGBoost is applied at the site scale to estimate the aggregated MAR-response and the decay coefficient. XGBoost was selected for these site-level predictions due to its ability to represent nonlinear relationships (Chen and Guestrin 2016) and its compatibility with model interpretation techniques. The complete list of input features for both models and their derivation are presented in “ML data preparation” section, and a summary of model targets is provided in Table 1.

#### ML data preparation

U-Net is trained on two-dimensional representations of the geo-hydrological properties from the numerical groundwater model, AMIGO. Following Fernandes et al. (2024), six inputs were shortlisted to capture the interactions within the system, applied recharge rate, aquifer transmissivity, vertical resistance below the aquifer, steady state groundwater depth, drain and river conductance, and groundwater depth below the drain level and river stage (shown on left-hand side of Fig. 1B). Among these inputs, the aquifer properties were further pre-processed as the 15 model layers were often discontinuous, represented by constant values (Vreugdenhil 2021; Fernandes et al. 2024). The model layers (shown on left-hand side of Fig. 1A) were merged into aquifers based on the vertical resistance: layers with resistance less than 200 days were combined, resulting in seven aquifers. Because artificial recharge was applied to the phreatic aquifer, only the properties of the first aquifer were used as model inputs. This aquifer comprises all 15 model layers in the east and the top four layers towards the west.

The vertical resistance below this aquifer was recalculated to include the cumulative resistance of the constituent layers. At locations where the aquifer comprises of all 15 model layers, the resistance of the basement is represented by the highest resistance between model layers. The resulting aquifer properties were then log-transformed and min-max normalized (0-1) to improve ML model training stability and performance. In contrast, the groundwater depth was not scaled or transformed as it is linearly related to the maximum MAR-response, and the direct drainage of excess recharge.

XGBoost requires tabular inputs, requiring manual feature extraction from the spatially distributed geo-hydrological properties used to train U-Net. In addition to capturing conditions at the MAR site, the extracted features need to represent surrounding properties that could impact the groundwater response. To satisfy this condition, the geo-hydrological properties around the site are summarized using a Gaussian kernel, which captures a distance-weighted average of the inputs (as shown in the central part of Fig. 1B). The weights are calculated based on the standard deviation of the Gaussian. After comparing standard deviations from 250 m to 1500 m, a value of 500 m trained the model with the highest R^2^. Based on this, a kernel with a standard deviation of 500 m is used for all the inputs. Some inputs are not defined everywhere, especially those for the surface water network. As an example, the river conductance is defined only at river streams, where the RIV package of MODFLOW is active. Locations where the RIV is inactive are represented by zero conductance before the Gaussian kernel is applied.

The Gaussian kernel is applied to all inputs to the XGBoost model, which are categorized into three groups, site properties – recharge rate, area of the recharge site; aquifer properties – transmissivity, vertical resistance, specific yield, and groundwater depth; and surface network properties – river density, river conductance, and drain conductance. The site properties are represented in U-Net by a map of the applied recharge, which represents both the recharge rate and the area (size) of the MAR site. These two inputs are provided separately to XGBoost.

For aquifer properties, the inputs include transmissivity, vertical resistance, specific yield, and groundwater depth for the situation without MAR (baseline scenario). The transmissivity and the vertical resistance undergo the same pre-processing as those used for the inputs to U-Net. While groundwater depth is not strictly an aquifer property, it results from the interaction of various location specific factors, such as transmissivity and seepage flux to the surface water network. Due to the localized nature of these influences, groundwater depth is included along with other aquifer properties. The groundwater depth calculated from the baseline scenarios, with only natural recharge, is divided into two periods: winter and summer. These periods were defined to coincide with the periods with and without recharge in “MAR scenarios” section. As the artificial recharge is applied during the winter months, the MAR-response is more strongly influenced by the winter conditions. On the contrary, the decay coefficient depends on summer conditions and is therefore predicted using the average summer groundwater depth.

In addition to the listed inputs, the transient phreatic groundwater heads are also dependent on the specific yield of the aquifer, unlike for steady-state groundwater heads which was the focus in Fernandes et al. (2024). For this study, the specific yield from the baseline scenario is used as an input. This value was computed for each time step by the unsaturated zone model MetaSWAP, which accounts for temporal changes in antecedent water content (Van Walsum and Groenendijk 2008). The time series of specific yield was divided into two periods: summer and winter, just like the groundwater depth data. The average winter specific yield is used to predict the MAR-response, while the average summer specific yield is used for the decay coefficient. The specific yield around the sites is represented using a Gaussian kernel, just like the other inputs.

Among the surface water network properties, the river and drain conductance are included as inputs in both XGBoost and U-Net models. In addition, river density is included as an input in XGBoost to quantify local drainage intensity. This metric was derived from a binary raster indicating active RIV cells (1 for active, 0 for inactive), to which a Gaussian kernel was applied. The resulting distance-weighted average produces a continuous, unitless river density metric that reflects both the proximity and concentration of river cells around each MAR site. Ditch distance is a common alternative for capturing similar information, which is calculated from river conductance. However, as river density is less dependent on the length of the river within the cell, it exhibits a weaker correlation with river conductance. This reduced correlation and the fact that river density is bounded between 0 and 1 result in more stable model training, making river density a more robust parameter for quantifying drainage intensity, especially for ditches and streams that are more than 25 m (numerical model resolution) away from each other. Finally, the groundwater depth relative to the drain level and river stage was excluded from the inputs to XGBoost as it was highly correlated with groundwater depth. Strong correlation complicates input attribution without significantly improving the model’s performance.

#### Training process

U-Net is trained iteratively with the ADAM optimizer with a reducing learning rate schedule with a relatively small batch size of 12. These hyperparameters have been shown to effectively train the model (Fernandes et al. 2024). Hyperparameters are predefined settings in a ML model that controls the learning process, influencing how the model optimizes its predictions and generalizes to new data. The model was trained on the results from 500 MAR sites and its performance was tracked during training using an unseen dataset, validation set, of 110 sites. The training iterations were stopped if the model performance on the validation set did not improve over ten consecutive epochs. The test set (110 sites) was held out entirely and not used during training or model optimization.

On the other hand, decision trees are added sequentially in XGBoost, and not iteratively, according to the chosen optimization metric and the predefined hyperparameters (such as such as tree depth, minimum node size, and learning rate). These hyperparameters were selected using a systematic procedure designed to maximize out-of-sample performance and generalizability. The results of all the scenarios are divided into training (N = 500) and testing sets (N = 220), which are used to train and assess the model’s generalization respectively. Employing repeated 10-fold cross-validation with 5 repeats, the model hyperparameters are tuned systematically, exploring a range of parameters through a grid search methodology. 500 values of each hyperparameter are sampled with Latin Hypercube Sampling. This rigorous evaluation across multiple folds mitigates overfitting and enhances reliability (Soper 2021). The hyperparameters with the highest cross-validation R^2^ were used to train the final model on the entire training set resulting in a robust XGBoost regression model improving the model’s generalizability to out-of-sample scenarios.

#### Interpreting the learnt relations

SHAP (SHapley Additive exPlanations) values provide a comprehensive method for interpreting ML models by attributing importance values to each input feature (Lundberg and Lee 2017; Young 1985). These values, derived from game theory principles, delineate the impact of individual features on model predictions. Here Tree SHAP is used (Lundberg et al. 2020), which is optimized for tree-based ML models such as XGBoost. Positive SHAP values indicate that a feature increases the predicted target, while negative values indicate a decrease. Importantly, the sum of all SHAP values for each feature equals the predicted target variable, as expressed in Eq. 4:

4 $$\begin{aligned} \hat{y} = \hat{y_0} + S_{x_1} + S_{x_2} + \dots + S_{x_n} \end{aligned}$$

where $\hat{y}$ is the predicted target, $\hat{y_0}$ is the average target across the training dataset, and $S_{x_1}$, $S_{x_2}$, ... $S_{x_n}$ are the SHAP values of the input features.

In this study, the target variables (MAR-response and decay coefficient) are log-transformed before training the model and the SHAP values explain the predictions on the log-transformed scale (see “ML data preparation” section and Fig. 3). To interpret the SHAP values in the original scale of the target variables, they are inverse-transformed by exponentiation. As per the product rule of exponents, this transformation converts the SHAP values from being additive to multiplicative, as shown in Eq. 5. In this transformed context, $10^{\hat{y_0}}$ approximates the average target value in the original scale. Here, a SHAP value of 1.5 indicates that the feature increases the prediction by 1.5 times, or 50% higher than the average target value $10^{\hat{y_0}}$.

5 $$\begin{aligned} 10^{\hat{y}} = 10^{\hat{y_0}} \cdot 10^{S_{x_1}} \cdot 10^{S_{x_2}} \cdot \dots \cdot 10^{S_{x_n}} \end{aligned}$$

### Application – identifying optimal regions

The trained ML models were applied across the entire domain to estimate the MAR-response and identify locations most suitable for MAR, in terms of the extra groundwater volumes stored and the decay of this stored groundwater with time. To maintain comparability, the MAR site area and rate were fixed at 10 ha and 15 mm day^-1^, respectively. In total, 1.6 million hypothetical MAR sites were evaluated, spaced 25 m apart to match the resolution of the geo-hydrological data.

Site performance was assessed using three metrics: (1) the steady-state MAR-response, (2) the transient MAR-response, and (3) the fraction of the MAR-response remaining after 3 months without recharge. The steady-state MAR-response was estimated using the U-Net model from Fernandes et al. (2024) , while the transient (5-month) MAR-response was predicted using XGBoost, which provides faster estimates than U-Net. The fraction of MAR-response was not directly predicted but derived from the decay coefficient estimated by XGBoost (Eq. 3). This formulation demonstrates the versatility of the decay coefficient, which is independent of duration without MAR and allows users to specify any dry-period length of interest.

## Results

This section first presents the estimated MAR-response, followed by the decay coefficient and their respective analyses. “MAR-response” section examines the MAR-response: (1) a comparison of steady-state and five-month transient estimates, (2) an evaluation of U-Net and XGBoost performance, and (3) an analysis of the key drivers of XGBoost predictions. “Decay coefficient” section focuses on the decay coefficient, which is defined only under transient conditions; accordingly, no steady-state comparison is provided. It then reports model performance and input-influence analysis. “Application – identifying optimal regions” section demonstrates the application by identifying optimal regions for MAR. Throughout this section, results generated by the ML models are referred to as ‘predicted’ while those from the numerical groundwater model are referred to as simulated. ML models are referred to as the trained/surrogate model, or by name: U-Net, XGBoost. Wherever the numerical model is used, it is explicitly named as such; otherwise, references to “model” pertain to the ML models.

### MAR-response

#### Numerical model simulations: transient scenarios

The simulated MAR-response and decay coefficient show skewed, approximately log-normal distributions across the 720 hypothetical MAR sites (Fig. 3). Most MAR-responses range between 0.21 and 2.3 million cubic meters (M m^3^) (10^th^ and 90^th^ percentile), with a median around 0.96 M m^3^, but a small number of sites exhibit much lower values. While the log-transformed MAR-response and decay coefficient approximates a normal distribution, MAR-response still retains slight skewness, particularly in the left tail.

#### Numerical model simulations: steady-state compared to transient scenarios

**Fig. 4:**
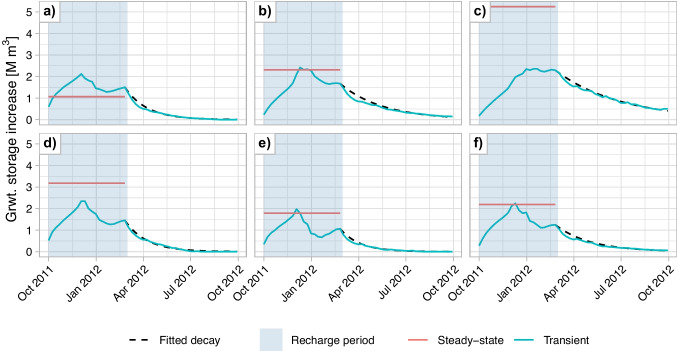
Groundwater response simulated at six hypothetical MAR sites. The steady state and the transient response are calculated from scenarios simulated with the numerical groundwater model AMIGO while the fitted decay is fit using a robust linear regression

**Fig. 5:**
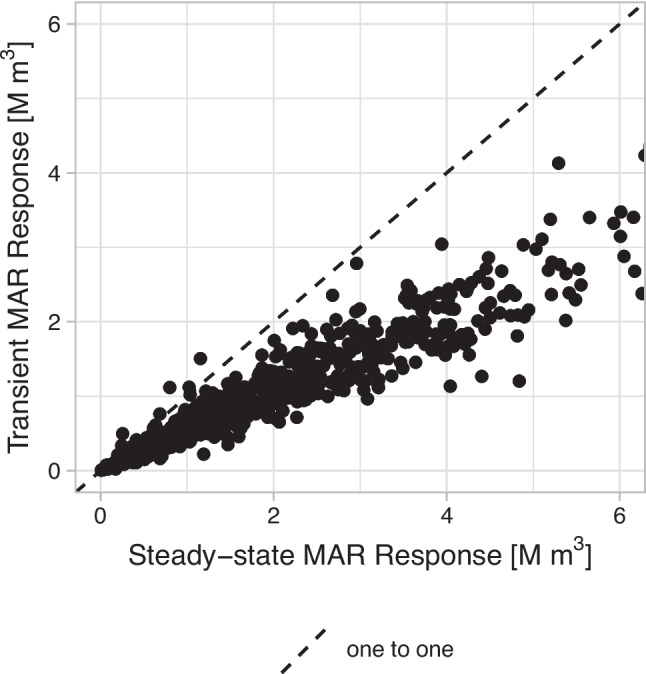
The volume of Steady-state response as compared to the transient MAR-response on 25th February, after five months of artificial recharge. Both responses are estimated using the numerical groundwater model. The dotted line indicates the 1:1 reference

When estimating the MAR-response, the groundwater response at the end of the recharge period, the steady state response could be a rather quick estimate that is simulated using the numerical groundwater model. It represents the result of long-term artificial recharge. However, seasonal variations and the short recharge period make this estimate inaccurate. The steady-state response typically exceeds the transient MAR-response for the majority of MAR sites, as illustrated in Figs. 4b-f and 5. On average, the transient MAR-response was 56% of the steady-state response, between 37% and 79% for 90% of the sites. Additionally, 10 sites exhibited a higher MAR-response than the steady-state response, one of which is depicted in Fig. 4a. This can be attributed to lower groundwater heads in February, near these MAR sites, compared to steady-state conditions due to the high transmissivity and proximity to streams and rivers. However, the transient MAR-response shows a strong correlation with the steady-state response, with an R^2^ of 0.92. This suggests that the steady-state response can serve as a good proxy to identify locations where MAR is likely to result in a high MAR-response. However, steady-state estimates do not provide precise quantification of the response, nor do they offer insights into the duration for which the stored water will remain in the subsurface. Addressing these questions requires simulating transient scenarios.

#### Performance of ML surrogates (XGBoost and U-Net)

XGBoost predicts the volume of the transient MAR-response based on the recharge rate applied at the site and averaged geo-hydrological properties around the site, as described in “ML data preparation” section. The MAR-response typically falls within 80% to 138% (10^th^ and 90^th^ percentile) of the response simulated with the numerical model. XGBoost is more accurate at predicting low MAR-response while underestimating high response (Fig. 7b). This could be due to the log transformation of the MAR-response before training the XGBoost model. Transforming the response helps reduce the bias in the trained model due to the right skew in the distribution of the MAR-response from the simulated scenarios. Even though the predictions show bias, XGBoost performs well overall, achieving a Mean Absolute Error (MAE) of 0.2 M m^3^, a Mean Absolute Percent Error (MAPE) of 17.84% with a coefficient of determination (R^2^) of 0.88.

U-Net predicts the response in each cell in the catchment based on 2-D inputs of geo-hydrological properties. Unlike the Gaussian kernel used to represent the properties around the MAR sites for XGBoost, U-Net learns to extract relevant features of the inputs during the model training process. The MAR-response simulated by the numerical model ranges between 82% and 164% of the predicted response by U-net. However, U-Net tends to underestimate low responses, as observed in four of the 120 sites tested. When focusing on sites with responses greater than 0.25 M m^3^, the upper bound of the range decreases significantly from 164% to 135%, indicating a better accuracy for large responses. Despite these occasional inaccuracies, particularly for low responses, U-Net achieves better overall performance than XGBoost, with a MAE of 0.16 M m^3^, an MAPE of 18.84%, and an R^2^ of 0.93, see Fig. 7a.

#### Key drivers of XGBoost predictions

**Fig. 6:**
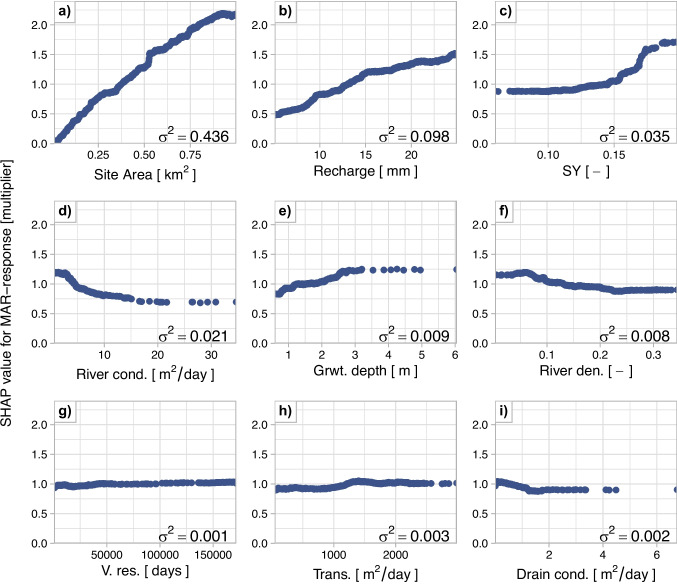
SHAP values of the inputs to the XGBoost model trained excluding specific yield from the inputs. SHAP values > 1 indicate that the feature at the MAR site contributed to a higher MAR-response compared to the average MAR-response for all training sites. Conversely, SHAP values < 1 indicate that the feature decreased the MAR-response. The variance of the SHAP values (annotated in the plots) indicates the importance of the inputs

Feature importance in the XGBoost model is indicated by the variance of SHAP values across all predictions, which is annotated as text in Fig. 6. Among the inputs, MAR site area and recharge rate are the dominant predictors of MAR-response consistent with expectations. Among the geo-hydrological properties, surface water network, specific yield, and groundwater depth explain most of the variation in the MAR-response. Transmissivity, vertical resistance and drain conductance have the least impact on the MAR-response. Furthermore, all inputs are positively correlated with the MAR-response except for the surface water network properties: river density, river conductance, and drain conductance, as shown in Fig. 6d, f, and i. This is consistent with the expected influence of the drainage network on MAR-response.

The SHAP values of the MAR-response show a near-linear relation area and recharge rate of the MAR site (Fig. 6a-b). These parameters affect the total recharge volume applied. However, the increase in the response diminishes for sites larger than 0.5 km^2^ or with recharge rate beyond 15 mm/day (Fig. 10a-b in the Appendix). Similarly, increasing the recharge rate beyond 15 mm/day shows a decreasing benefit in the response (Fig. 10b). This nonlinearity suggests distributing recharge across multiple smaller sites may store more water overall than concentrating it in a single large site.

Specific yield appears highly influential in the SHAP analysis: values > 0.17 relate to +75% MAR-response, and decreases from +50% to -12% between 0.10 and 0.17. However, this trend lacks physical basis, as higher specific yield should result in smaller head increases for the same recharge. The effect likely reflect multicollinearity between specific yield, river density, and groundwater depth (R^2^ > 0.65), complicating the interpretation of individual effects (see Figs. 11, 12, and 13 in the Appendix). Part of the surface drainage driven influence is attributed to specific yield.

Following the site design parameters and specific yield, the properties of the surface water network have the biggest and a nonlinear impact on the MAR-response (Fig. 6c, e and h). Sites with very low river density show a maximum of 20% higher response and reduces linearly between river density of 0.05 and 0.225 (unitless) to 10% below the average MAR-response (i.e. SHAP = 1). Similarly, MAR-response decreases exponentially with increasing river conductance: sites near channels with conductance below 2 m^2^/day show up to 20% higher responses, whereas those near major rivers with conductance above 15 m^2^/day exhibit about 30 % lower responses due to greater drainage capacity. Tile drainage has a comparatively minor influence, varying between roughly +5% and -10%, though its limited spatial extent reduces confidence in this relation. Overall, these patterns suggest that high drainage intensity is undesirable for maximizing MAR-response.

The groundwater depth is a unique input feature as it is the result of groundwater flow that is determined by multiple system characteristics, such as river density, river stage, and transmissivity. It also determines the available storage space for the MAR-response, acting as a limiting factor when groundwater levels are within 3 m (Fig. 6e). The MAR-response at these sites varies between 15% lower to 25% higher than the average predicted MAR-response. In areas with deeper groundwater levels, groundwater depth has minimal impact on the MAR-response.

Among the inputs compared, the aquifer properties have the smallest impact on the MAR-response (Fig. 6g and h). MAR-response increases with transmissivity, from about 5 to 10% lower response at values less than 1000 m^2^/day to 5% higher response for more transmissive aquifers, up to 1300 m^2^/day (Fig. 6h). Vertical resistance has a minor but highly non-linear impact on the response (Fig. 6g), with low vertical resistance related to a smaller MAR-response, up to 8% lower response, and high resistance resulting in a high MAR-response, up to 8% higher.

### Decay coefficient

#### Numerical model results: transient scenarios

The simulated decay coefficients are concentrated between 0.0086 day^-1^ and 0.047 day^-1^, with a median around 0.022 day^-1^, with few sites showing much slower or faster drainage, see Fig. 3b. A decay coefficient of 0.0073 day^-1^ is rarely achieved in the simulation dataset. For reference, a decay coefficient of 0.0073 day^-1^ represents a case where 80% of the stored water drains within the 219 days without MAR. Of the 720 randomly selected hypothetical MAR sites used to train the model, only 52 have a lower decay coefficient. While some of these sites are reasonably efficient for long-term storage, identifying them requires a targeted approach. This highlights the importance of optimization in identifying site locations better suited for artificial recharge.

#### Performance of ML surrogate (XGBoost)

**Fig. 7:**
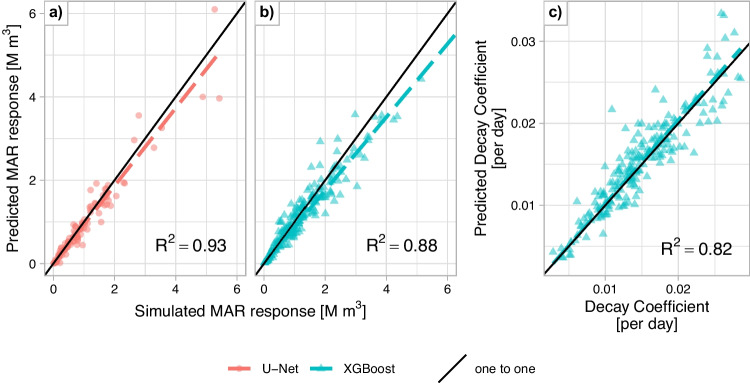
Simulated versus predicted values for key Managed Aquifer Recharge (MAR) metrics at test MAR sites used to evaluate ML models. Panels a and b show MAR-response predictions, while Panel c shows predictions of the decay coefficient. In all panels, a colored dashed line represents the linear trend, and a solid black line indicates the 1:1 reference line

XGBoost can accurately predict the decay rate of the simulated response. The simulated decay rate is within 82% to 122% of the predicted decay rate (10^th^ to 90^th^ percentile), while not showing a systematic bias, as evidenced in Fig. 7c. The MAE of the trained model is 0.0022 day^-1^, MAPE is 13% and an R^2^ of 0.82. Relative to the MAR-response, the decay coefficient proved to be more difficult to predict. The model has a lower R^2^, but also a lower MAPE suggesting that while the trained model does not explain all the variance in the data, it is more accurate at predicting low decay coefficient resulting in a lower MAPE. This combination suggests that the trained XGBoost models are especially accurate at identifying efficient MAR sites, with high MAR-response and low decay coefficient.

#### Key drivers of XGBoost predictions

**Fig. 8:**
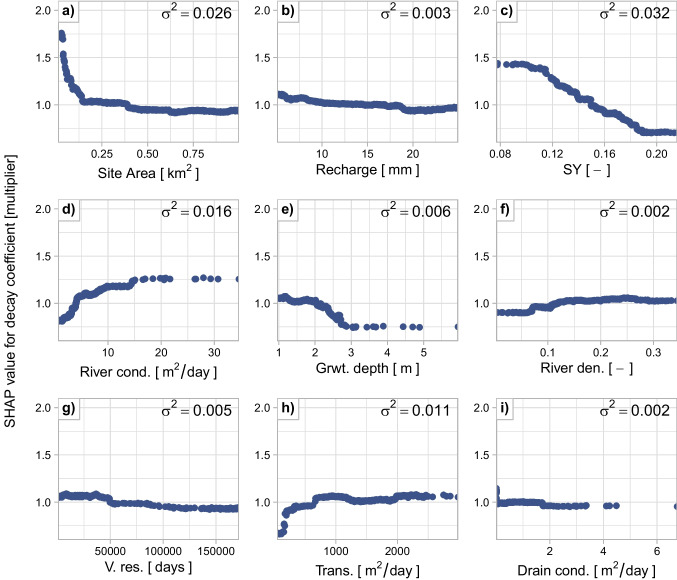
SHAP values of the inputs to the XGBoost model trained excluding specific yield from the inputs. SHAP values > 1 indicate that the feature at the MAR site contributed to a higher decay coefficient compared to the average decay coefficient for all training sites. Conversely, SHAP values < 1 indicate that the feature slowed the decay of the response. The variance of the SHAP values (annotated in the plots) indicates the importance of the inputs

The most informative inputs for predicting the decay coefficient differ from those that influence the MAR-response. Site area and recharge rate, have a limited influence on the decay coefficient (Fig. 8a-b), except for very small sites (area < 0.1 km^2^), where the decay rate increases sharply. This behavior increases the variance in SHAP values for site area, making it appear as the second most important input after specific yield. This pattern suggests that smaller sites are more strongly affected by their surroundings than larger sites.

Sites with a high specific yield have a low decay coefficient (Fig. 8c) consistent with high specific yield buffering change in groundwater head to fluxes. XGBoost identified this relation, learning an approximately linear decrease in decay coefficient from about +40% to -25% between specific yield values of 0.10 and 0.19. However, the constant SHAP values at the extremes are likely an artifact of the tree-based model and the sparse data in those ranges. The linear trend would likely continue if the model were trained with more sites at these extremes.

The surface water network is the third most important input for predicting the decay coefficient. As river conductance increases from 0 to 15 m^2^/day, the decay coefficient rises from about -20% to +20%, and up to +25% for higher conductance values (Fig. 8d). River density and drain conductance are less influential but show similar patterns: low values correspond to decay coefficients up to ~10% lower than average, while sites with densities above 0.15 show a modest increase of up to 2.5% (Fig. 8f). Drain conductance also has a minor effect but follows a similar trend, probably due to its poor representation in the region. Together, these inputs represent drainage intensity around each site, where higher intensity leads to faster depletion of stored water, an effect amplified in this study area by shallow groundwater levels.

Aquifer properties, specifically transmissivity, groundwater depth, and vertical resistance, are the fourth to sixth most important inputs for predicting the decay coefficient. Low transmissivity is associated with lower decay rates (Fig. 8h), whereas values above 700 m^2^/day correspond to decay coefficients that are 5% to 12% higher than average. Groundwater depth also shows a clear trend: decay decreases roughly linearly between 2 and 3 m and then plateaus at deeper levels (Fig. 8d). Vertical resistance likewise influences the decay rate. Regions underlain by a single aquifer, exhibit decay coefficients 5% to 10% below average, while, areas with multiple aquifers have decay coefficients 5% to 10% above average (Fig. 8g).

### Application – identifying optimal regions

**Fig. 9:**
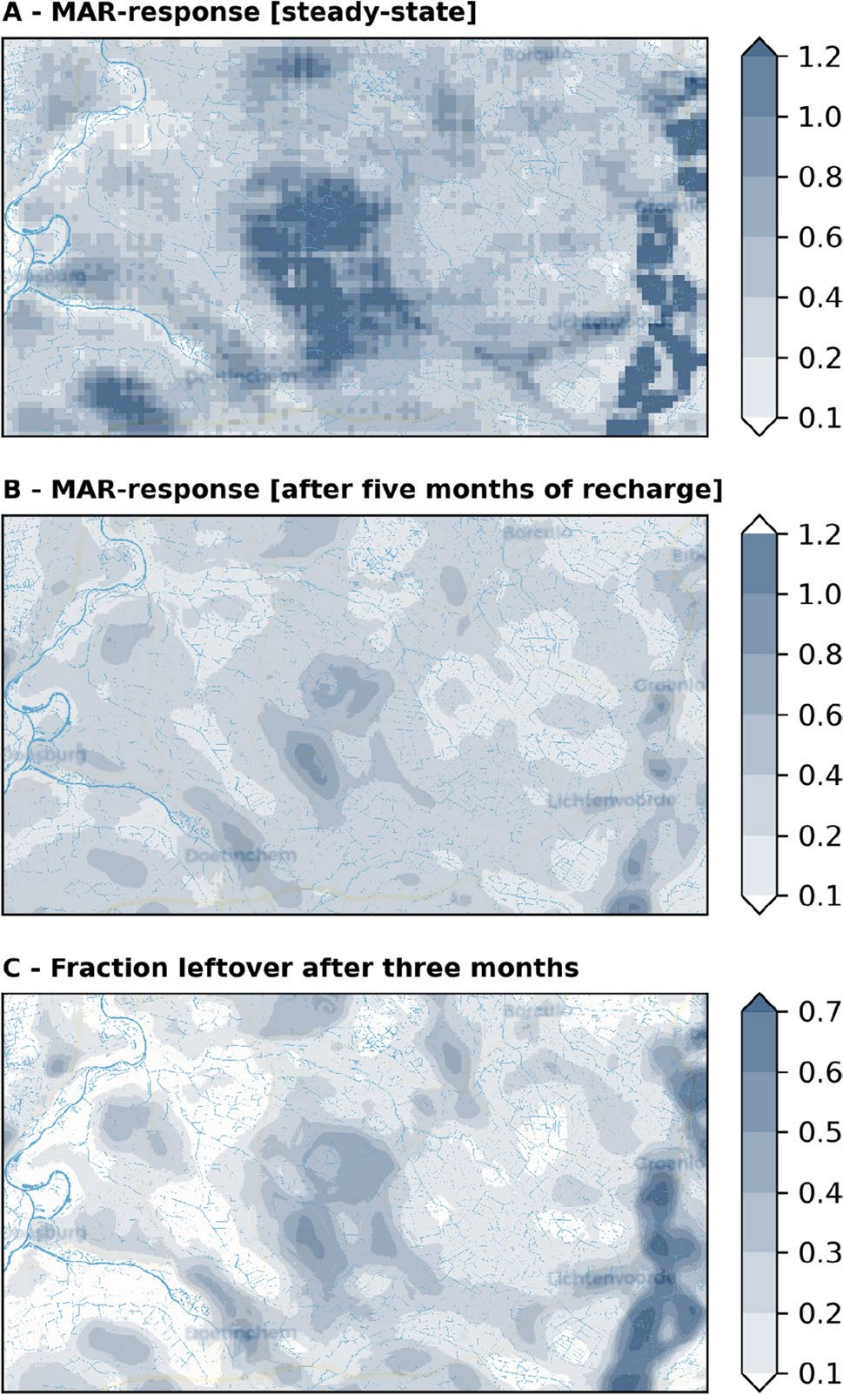
The groundwater response to 15 mm/day of artificial recharge applied over 10 ha MAR sites. Panel a shows the steady-state MAR-response in million m^3^ as estimated using U-Net (Fernandes et al. 2024). Panel b presents the MAR-response after five months of recharge, also in million m^3^ is estimated by XGBoost. Panel c displays the fraction of water remaining after three months [unit less], calculated from the decay coefficient estimated XGBoost. The surface water network in the region is indicated by blue lines

**Fig. 10:**
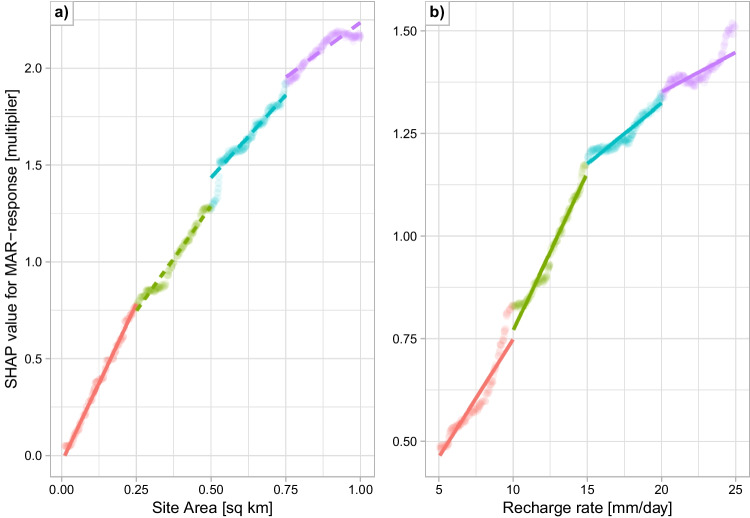
The effect of MAR site area and the recharge rate applied at the site on the predicted MAR-response. Four trend lines are drawn through the points for each quantile to emphasize the changing slope across the range

**Fig. 11:**
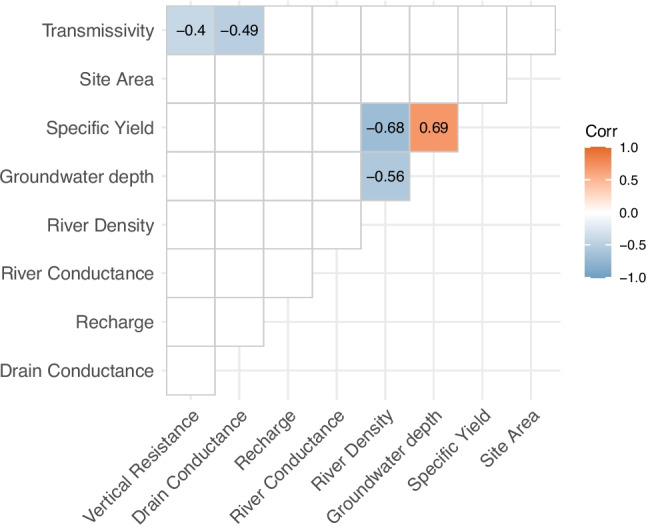
Correlation matrix of all pairs of input variables to XGBoost. Only statistically significant correlations (p-value < 0.05) are shown

**Fig. 12:**
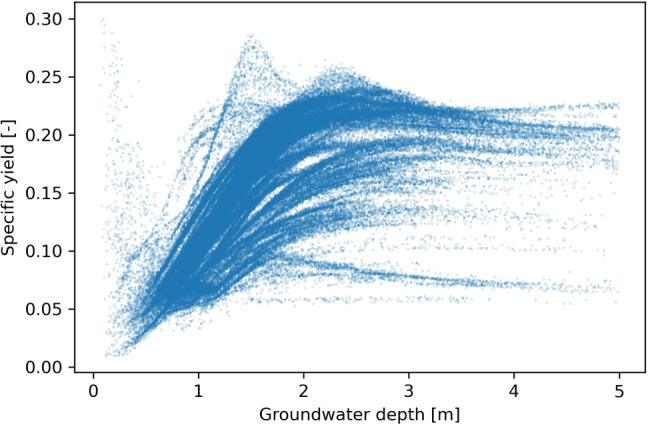
The relation between the specific yield and the groundwater depth, as a scatter plot. The specific yield is modeled by the unsaturated zone model MetaSWAP. The specific is lower for locations with a deeper groundwater which leads to a higher specific yield

**Fig. 13:**
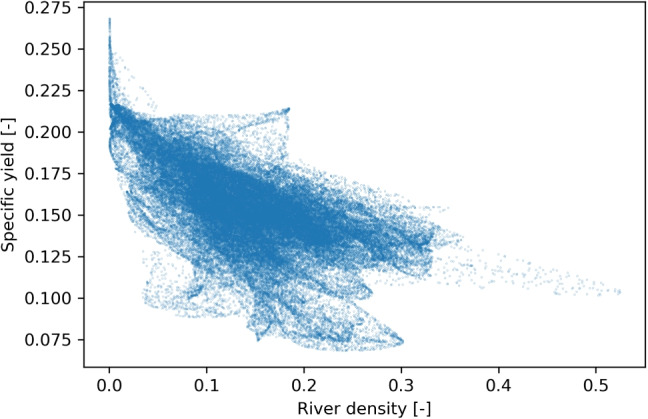
The relation between the specific yield and the river density, as a scatter plot. The specific yield is modeled by the unsaturated zone model MetaSWAP while the river density is calculated from the inputs to the numerical groundwater model. The moisture content is lower for locations with a deeper groundwater which leads to a higher specific yield

The trained ML models, being computationally efficient, can be used to estimate the groundwater response to MAR sites across the entire study area, creating a regional scan of potential MAR sites, Fig. 9. The two characteristics of the groundwater storage were estimated at 1.6 million hypothetical MAR sites that were spaced at 25 m between their centers. These estimates were completed within 20 seconds, whereas the equivalent numerical simulations would require roughly 1000 CPU-years, representing a speed-up of nine orders of magnitude. The surface network has a strong influence on the MAR-response and the fraction of the response left over after 3 months. Both the targets are reduced around the rivers to the west and the south-west, except at the higher regions just north of the south-western river due to the relatively higher elevation in the region.

The area to the east especially shows a high potential for MAR, as recharged water stays within the groundwater system for longer due to the low transmissivity of the aquifer in this region. The area in the center of the catchment shows a comparable initial MAR-response, but retains a smaller fraction of water after three months, making the area less suitable for MAR. While the steady-state response correlates well with the MAR-response, these results also show that a transient approach is needed, as areas that might look promising from a steady-state approach, appear to be less suitable in the more realistic transient case.

## Discussion

The findings presented above suggest that a ML surrogate can effectively estimate the increase in groundwater storage due to seasonal artificial recharge. The groundwater storage reaches a maximum during the recharge period after which it decays back to the natural groundwater levels. Leveraging this relationship enabled the decomposition of the time series into two components, the MAR-response and the decay coefficient, which simultaneously simplifies modelling and allows prioritizing either component based on specific MAR objectives. The flexibility to prioritize either component is a special advantage of using transient scenarios over steady-state scenarios. One possible optimization task is demonstrated in “Application – identifying optimal regions” section, where we estimate the fraction of the groundwater storage remaining five months after MAR was stopped, along with the MAR-response at the end of the recharge period. These estimates were generated for 1.6 million hypothetical sites within seconds, highlighting the computational efficiency of surrogate models. This speed enables more thorough exploration of possible MAR sites and makes it feasible to integrate transient behavior into early-stage decision-making and optimization workflows.

### ML modelspretability-accuracy’ complexity-interpretability-accuracy

Among the two ML techniques for estimating the MAR-response, U-Net provides the most accurate estimate. It boasts a higher R^2^ value and a lower MAPE. This better performance could be explained by U-Net’s ability to extract high-level features from the inputs in the encoder. These features are not as physically interpretable as the Gaussian kernel used to represent the features around the MAR site as used in XGBoost. Furthermore, the Gaussian kernel is agnostic to the orientation of features such as those of the surface water network. The MAR-response would be higher if all streams were on the same side of the MAR site than if the streams surrounded the site. A feature extraction that can capture this relation could help improve XGBoost. Techniques such as Gabbor filter, Fourier transform, and wavelet transform could help better describe the density and orientation of the surface water network. However, these filters extract multiple representations, such as multiple frequencies in the case of Fourier transformation, which would complicate model interpretability. So, there is a trade-off to be made between the model complexity and the interpretability of the ML model. Despite this disadvantage, the Gaussian kernel has proved to adequately represent the properties around the site while also maintaining a physical interpretation of being the distance-weighted average which can be used to define a range of influence of the feature on nearby MAR sites. Furthermore, as a tree-based model, XGBoost is also more interpretable than U-Net, which facilitates identifying and quantifying the effect of each of the inputs on the MAR-response based on the SHAP values of the model. All identified relations can be explained based on known theoretical relations and the identified collinearity between the input features.

### Efficient size and recharge rate of the site

MAR site design decisions have the strongest influence on the expected response of a system based on the SHAP values from XGBoost. A high recharge rate applied over a large MAR site can effectively cause a high MAR-response. While this is consistent with expectations, the efficiency of the design decision cannot be commented on without simulations. XGBoost identified that the efficiency of the MAR site reduces for very high recharge rate suggesting multiple small sites would be more efficient than a single large MAR site. Conversely, the groundwater storage due to very small MAR sites decays faster than larger sites. Overall, multiple MAR sites larger than 0.1 km^2^ each recharging up to 20 mm/day would offer the largest volume of increased storage in the groundwater.

### Inaccurate effect of groundwater depth

Furthermore, the observed relationship between groundwater depth and MAR-response appears to level off beyond 3 m, with only a minor effect on the decay coefficient. However, this likely reflects the characteristics of the study area, where groundwater lies within 3 m of the surface across 98% of the catchment, rather than a true change in the underlying relationship. It is plausible that, if the model were trained on sites with greater groundwater depths, the linear trends observed would continue, for both MAR-response and decay coefficient. This suggests that, in regions with deeper groundwater, artificial MAR sites may support greater storage potential and slower drainage, making them preferable for long-term retention.

### Effect of correlated inputs

This is especially crucial as favorable site properties can substantially increase the expected groundwater storage, with specific yield, groundwater depth, and the surface water network playing a particularly vital role. These factors are also strongly correlated, which reduces confidence in isolating their individual effects. Nevertheless, their relative importance suggests that they also contribute independently to overall site suitability. In highly managed landscapes, such as in the Netherlands, strategically reducing the number, width and depth of small streams can decrease the river density and conductance, potentially improving site suitability for long-term groundwater storage.

### Effect of aquifer properties

Among the aquifer properties, locations in a more transmissive aquifer with deep groundwater are commonly identified as potential MAR sites (Gibson and Campana 2014; LaHaye et al. 2021). These relations are based on Brown et al. (2005) who determined 450 m^2^/day to 2300 m^2^/day to be optimal for aquifer storage and recovery at MAR sites across South Florida. A lower transmissivity could lead to significantly high heads at the MAR site, mounding, while the upper limit maximizes recovery of the stored water, which is outside the scope of this study. While a similar relationship is observed between high transmissivity and MAR-response, transmissivity of less than 750 m^2^/day is particularly conducive to long-term storage of the water. Overall, aquifer transmissivity has a minor impact on groundwater storage relative to the influence of the surface water network.

### Unexpected effect of specific yield

Although specific yield is traditionally considered insensitive for aquifer storage and recovery (Brown et al. 2005; Merritt 1986; Yobbi 1996), it emerged as one of the most influential inputs in this analysis. It significantly affects the predictions of both MAR-response and decay coefficient. A high specific yield is also related to a high MAR-response, but this relation lacks a theoretical explanation. This would suggest that multicollinearity between groundwater depth, river density, and specific yield has influenced the relation. However, as specific yield affects how quickly the groundwater heads react to fluxes in the groundwater, it also affects the rate at which the groundwater decays to the surface water network.

### Advantages of SHAP explanations

Surrogate modelling enabled a more robust attribution of feature impact than would have been possible using traditional sensitivity techniques. The variant of SHAP values used in this study, Tree-SHAP, is computationally efficient and provides more accurate Shapley value attributions for tree based models, making it well-suited for dataset-wide interpretations (Lundberg and Lee 2017; Aas et al. 2021). Compared to TreeSHAP, traditional global sensitivity analysis methods such as Sobol’ indices provide a variance-based decomposition of model output rather than local explanations of individual predictions. This formulation makes it highly sensitive to input correlations, requiring specific adaptations under dependence, and their interpretation becomes non-unique. In contrast, Tree-SHAP adheres to the key axioms of Shapley values of efficiency, symmetry, dummy, and additive, hence offering a more equitable allocation of each input’s contribution to the model output (Owen 2014; Iooss and Prieur 2019).

### Interactions between the inputs

While SHAP values can capture both main effects and interactions between features, this study focuses only on the prior to ensure generalizability across the region. Including interaction effects may offer deeper insight at the local scale, but such interactions are often specific to the training context and may not transfer reliably to other areas. When such interactions are of interest, a more appropriate approach would be to train a surrogate model directly on data from the region of interest, enabling both accurate predictions and meaningful interpretation of localized interactions through SHAP interaction values.

### Applying models to new regions

The relations identified in this study are especially applicable when optimizing the location and recharge rate at MAR sites in regions with intensive drainage networks and relatively shallow groundwater during part of the year. The properties of the regional hydrology would likely result in significantly different features being important as can be seen when comparing the findings of this paper with those from Brown et al. (2005). However, further research is required to test the generalizability of these findings across different regions, providing the evidence necessary to confidently accept the relations. However, being a tree-based model, XGBoost would likely underestimate the MAR-response and the decay coefficients where the geo-hydrological conditions are beyond the range in the training data. The model could be retrained on numerical model scenarios from these regions to improve the applicability of the model. Furthermore, increasing the diversity of the training data would also minimize overfitting.

### Implications for different climatic conditions

The results of this study are based on simulations using average meteorological conditions, specifically, a year representing the mean precipitation surplus for the catchment (see Fig. 2). Under wetter or drier conditions, MAR-induced groundwater storage is expected to change primarily through shifts in natural recharge and groundwater depth, which determine the conditions in the baseline scenario from the numerical groundwater model. Because MAR-induced storage is evaluated relative to this baseline, much of the direct effect of natural recharge is expected to cancel in the differencing. In contrast, groundwater depth, one of the ML model inputs, varies directly with climatic conditions, and the models were trained and evaluated across a wide range of groundwater depths. Shallower groundwater under wetter conditions would enhance seepage to the surface-water network, reducing the MAR-response and increasing the decay coefficient. This behavior is consistent with the SHAP-based relations for groundwater depth (Figs. 6e and 8 6e), suggesting that the XGBoost model captures the expected qualitative response. Nonetheless, the magnitude of this effect has not been evaluated in this study and could be verified under more diverse climatic conditions.

### Implication for shorter term storage

A simplification of this study lies in the use of a single decay coefficient to characterize post-recharge storage decline. The groundwater storage often declines in two phases, an initial rapid drop followed by a slower, more stable recession, particularly at sites with higher drainage intensity (see Fig. 4b and f). While this simplified metric may underestimate initial decline, it captures long-term behavior more effectively and is therefore appropriate for evaluating interannual impacts of seasonal MAR. The difference between initial and long-term decay is more pronounced at sites with high decay rates and can be significant when optimizing for shorter-term storage.

## Conclusion

This study demonstrates the potential of ML surrogate models to assess transient groundwater responses to MAR at high spatial resolution. U-Net and XGBoost effectively capture the dynamics, MAR-response and decay coefficient, affecting groundwater storage due to artificial recharge, based on the geo-hydrological properties of a location. While steady-state estimates can serve as a good proxy, they fail to capture all the variance in the response to MAR and provide limited insights into the long-term storage. In contrast, the trained ML models capture a majority of the variance, achieving good performance metrics with R^2^ higher than 0.8 and mean percent error below 20%.

Beyond predictive accuracy, a key advantage of the ML models is their computational speed, enabling rapid, region-wide assessment of MAR potential. Although the surrogate ML model does not replicate the full physical detail of the numerical model, XGBoost evaluated 1.6 million hypothetical sites in under 20 seconds, about nine orders of magnitude faster than numerical simulations, making domain-wide MAR assessment feasible. This efficiency makes it feasible to incorporate transient dynamics into early-stage planning, providing a practical and scalable tool for identifying sites with both high initial response and sustained storage potential.

Our findings show that different inputs influence the MAR-response and the decay coefficient, highlighting the need to evaluate both when selecting sites for artificial recharge. While site area, recharge rate, and specific yield mainly determine the total volume stored, long-term retention is more strongly controlled by specific yield, river conductance, and aquifer properties such as transmissivity and vertical resistance. These differences underscore that high storage potential does not necessarily imply sustained retention. This has direct implications for multi-criteria decision analysis (MCDA): given the variation in criteria used across studies, as noted by Sallwey et al. (2019), the results provide a data-driven basis for prioritizing inputs that matter most for both recharge efficiency and storage longevity.

Furthermore, the eastern sandy soils in The Netherlands offered unique conditions that made some of the commonly applied criteria for site selection less suitable. For example, the surface water network was found to hinder long term storage due to the region’s shallow groundwater, efficient drainage network, and highly permeable sandy aquifers which lead to high return flow. This finding differs from other studies where proximity to rivers is considered advantageous as a source of water for recharge. These results underscore the importance of tailoring site selection to the specific hydrogeological context, where ML surrogate models could play a vital role in guiding decisions and maximizing the effectiveness of the MAR interventions.

While this study identified important criteria influencing MAR-induced groundwater storage, their broad scale applicability is yet to be demonstrated. Expanding to a larger and more diverse training dataset (across hydro-geological settings and for different climatic conditions) would help the model capture the non-linear relations and improve generalizations. Moreover, interactions between the criteria may significantly alter their influence, follow-up work should explicitly analyze interactions and dependence before making strong conclusions based on the SHAP attributions identified in this study.

The surface water network is likely influential in regions with shallow groundwater. These networks also matter during periods of excess precipitation, particularly for agricultural areas. A comprehensive, multi-objective strategy, maximizing groundwater storage while mitigating wet-season impacts, is therefore needed and could be supported by the surrogate modelling in combination with explainable AI techniques like demonstrated here.

## Data Availability

The meteorological data is downloaded from KNMI Data Portal, and is available under Creative commons license 4.0; https://api.dataplatform.knmi.nl/open-data/v1/datasets/Rd1/versions/5/file, and https://api.dataplatform.knmi.nl/open-data/v1/datasets/EV24/versions/2/files. The numerical groundwater model used in the study is described in this paper Vreugdenhil (2021). The access conditions and requirements to run the scenarios are listed in the documents available in ‘AMIGO Dashboard’ https://experience.arcgis.com/experience/80d3bce11bca4694b53a9941a37d0030/page/AMIGO/?views=Info-. The scripts used to train and analise the models in the paper can be found at GitHub, https://github.com/ValdrichF/aqc6.3-transient-mar, and is preserved at 10.5281/zenodo.14582956 for the review process.
